# The Airway Microbiome as a Modulator of Influenza Virus Infection: Mechanistic Insights and Translational Perspectives—Review

**DOI:** 10.3390/pathogens15010063

**Published:** 2026-01-07

**Authors:** Georgia Gioula, Maria Exindari

**Affiliations:** Department of Microbiology, School of Medicine, Aristotle University of Thessaloniki, 54124 Thessaloniki, Greece; mexidari@auth.gr

**Keywords:** airway microbiome, influenza, interferon-λ, neuraminidase, proteases, short-chain fatty acids, *Staphylococcus epidermidis*, *Streptococcus oralis*, *Staphylococcus aureus*, *Streptococcus pneumoniae*, live biotherapeutic products, gut–lung axis

## Abstract

Outcomes of influenza virus infection vary widely across individuals, reflecting not only viral genetics and host factors but also the composition and function of the airway microbiome. Over the past few years, mechanistic work has clarified how specific commensals (for example, *Staphylococcus epidermidis* and *Streptococcus oralis*) restrict influenza replication by priming epithelial interferon-λ programs, reshaping intracellular metabolite pools (notably polyamines), dampening host protease activity, and maintaining barrier integrity; meanwhile, pathobionts (notably *Staphylococcus aureus* and *Streptococcus pneumoniae*) can enhance viral fitness via secreted proteases and neuraminidases that activate hemagglutinin and remodel sialylated glycoconjugates and mucus, setting the stage for secondary bacterial disease. Recent studies also highlight the gut–lung axis: gut microbiota-derived short-chain fatty acids (SCFAs), especially acetate, protect tight junctions and modulate antiviral immunity in influenza models. Together, these insights motivate translational strategies—from intranasal live biotherapeutics (LBPs) to metabolite sprays and decoy/dual neuraminidase approaches—that complement vaccines and antivirals. We synthesize recent evidence and outline a framework for leveraging the airway microbiome to prevent infection, blunt severity, and reduce transmission. Key priorities include strain-level resolution of commensal effects, timing/dosing windows for metabolites and LBPs, and microbiome-aware clinical pathways for anticipating and averting bacterial coinfection. Overall, the airway microbiome emerges as a tractable lever for influenza control at the site of viral entry, with several candidates moving toward clinical testing.

## 1. Introduction

Influenza viruses are major causes of seasonal respiratory disease and periodic pandemics, resulting in substantial morbidity and mortality worldwide. Although viral genetics and host immune history significantly shape disease outcomes, the airway microbiome has emerged as an important additional determinant of infection susceptibility, viral replication dynamics, and clinical severity [1,2]. The airway microbiome refers to the communities of bacteria, fungi, and viruses residing in the nasal cavity, nasopharynx, and lower respiratory tract. These microbial communities influence immune tone, barrier integrity, mucus composition, and epithelial antiviral signaling even in the absence of infection [3,4].

Early evidence for microbiome involvement in influenza came from antibiotic-treated mouse studies showing that depletion of commensal microbes reduced antiviral immune responses and led to more severe disease [5]. This finding led to the discovery that commensal-derived signals prime mucosal interferon pathways, particularly interferon-λ (IFN-λ) responses that are essential for antiviral protection in the upper airway [6]. More recently, *Staphylococcus epidermidis*, a common nasal commensal, has been shown to enhance IFN-λ signaling in nasal epithelial cells and restrict influenza virus replication in both cell culture and animal models [7,8]. Further mechanistic work has demonstrated that *S. epidermidis* can also modify epithelial polyamine metabolism, creating a cellular environment less permissive to viral replication [9].

In contrast, influenza destabilizes the airway microbiome by damaging epithelial barriers, impairing mucociliary clearance, remodeling mucus and sialylated glycans, and transiently suppressing antibacterial immune functions, thereby favoring loss of commensals and expansion of pathobionts, such as *Streptococcus pneumoniae* and *Staphylococcus aureus* [10,11]. These pathobionts can worsen influenza disease through several mechanisms. For example, pneumococcal neuraminidases remove sialic acids from mucin and epithelial glycoconjugates, altering influenza receptor landscapes and enabling bacterial adherence and invasion [12]. Meanwhile, *S. aureus* secretes proteases and lipases that can enhance influenza viral entry and promote necrotizing pneumonia in co-infection settings [13,14].

Another key layer of microbial–host interaction occurs through the gut–lung axis, in which gut microbiota-derived metabolites influence lung immune responses. Short-chain fatty acids (SCFAs), including acetate, have been shown to protect epithelial barrier integrity and reduce influenza-associated lung injury in mouse models [15]. These findings suggest that microbiome influences on influenza outcomes are systemic as well as local.

Together, emerging evidence indicates that the airway and gut microbiomes shape influenza infection by regulating antiviral immune priming, enzymatic environments that control viral entry and activation, and susceptibility to secondary bacterial complications. Increasing mechanistic clarity has positioned the microbiome as a promising therapeutic target for reducing influenza severity and transmission. The following sections examine these interactions in detail and outline translational strategies to leverage microbiome-mediated protection.

While prior reviews have described broad associations between the microbiome and influenza infection, the present review emphasizes recent mechanistic insights that move beyond correlation. Specifically, we highlight strain-level commensal effects on interferon-λ priming, epithelial metabolism, protease regulation, oxygen microenvironments, and barrier integrity at the airway mucosa. We also integrate these mechanisms with emerging translational strategies and clinical considerations, including antibiotic stewardship, safety of live biotherapeutics, and timing of microbiome-targeted interventions. Together, this approach reframes the airway microbiome as an actionable therapeutic interface at the earliest stages of influenza infection.

## 2. Composition and Function of the Airway Microbiome

The airway microbiome represents a structured microbial ecosystem that varies by anatomical region, host age, environment, and immune status. Unlike the gut microbiome, which is dense and dominated by obligate anaerobes, the airway microbiome is a low-biomass, oxygen-tolerant community shaped by airflow, mucociliary clearance, and continuous exposure to inhaled microbes. Though smaller in density, the airway microbiome has disproportionate functional influence over mucosal immunity and susceptibility to respiratory pathogens, including influenza virus [1,3].

### 2.1. Regional Organization and Dominant Microbial Taxa

The upper airway (including the anterior nares, nasal cavity, and nasopharynx) is typically enriched with Corynebacterium, Dolosigranulum, coagulase-negative staphylococci such as *Staphylococcus epidermidis*, and commensal Streptococcus species belonging to the viridans group [1,3,16]. These taxa are associated with mucosal homeostasis, maintenance of epithelial barrier function, and colonization resistance, which limits the ability of pathogens to gain a foothold.

The oropharynx reflects transitional overlap with the oral microbiome and includes taxa such as *Prevotella*, *Veillonella*, and *Actinomyces* [17]. The lower respiratory tract, once thought to be sterile, is now recognized to harbor sparse but persistent microbial communities acquired through microaspiration and shaped by immune surveillance [2,18]. In healthy lungs, microbial signatures often resemble those of the oropharynx but in lower abundance.

In infants and young children, the nasopharyngeal microbiome undergoes dynamic assembly, with *Dolosigranulum*–*Corynebacterium*–*Staphylococcus*–dominated profiles associated with protection against respiratory infection, whereas *Moraxella*- and pathogenic streptococcal dominance correlates with higher incidence of pneumonia and wheezing disorders [16,19]. These early-life microbial patterns influence later respiratory health and immune responses, suggesting a developmental window where airway microbiota “educate” mucosal immunity.

### 2.2. Stability and Perturbation of the Airway Microbiome

Airway microbial communities exist in a balanced ecological state maintained by host defenses (including mucus flow, antimicrobial peptides, and pattern recognition signaling) and microbe–microbe competition. This balance can be disrupted by infections, antibiotics, corticosteroids, pollution, smoking, and aging [4,20].

Influenza infection is a major driver of microbial perturbation. It disrupts airway microbial stability through several interrelated host- and virus-driven mechanisms. Viral replication damages the respiratory epithelium, impairs ciliary function, and alters mucus composition, collectively reducing mucociliary clearance and allowing opportunistic bacteria to persist and expand. Influenza-induced interferon responses and epithelial cell death reshape the local nutrient and oxygen landscape, favoring facultative anaerobic pathobionts such as *Streptococcus pneumoniae* and *Staphylococcus aureus*. In parallel, viral neuraminidase activity and host inflammatory enzymes remodel sialylated glycoconjugates on the epithelial surface and within mucus, altering microbial adhesion niches. Finally, influenza transiently suppresses phagocyte antibacterial function, creating a window in which dysbiotic communities can establish and persist even after viral clearance [10].

Studies in both humans and animal models show that influenza results in:

- Reduced microbial diversity
- Loss of beneficial commensal taxa (e.g., *Dolosigranulum*, *Corynebacterium*)
- Expansion of pathobionts, particularly *Streptococcus pneumoniae*, *Staphylococcus aureus*, *Haemophilus influenzae*, and *Moraxella catarrhalis*
- Altered mucus composition and ciliary function, facilitating persistent dysbiosis [10,11].

These changes may persist weeks after viral clearance, illustrating how influenza can reshape the airway microbial environment in ways that influence secondary infection risk and long-term respiratory outcomes [11].

### 2.3. Protective Commensals and Their Mechanisms of Action

Emerging evidence identifies specific commensal taxa that protect the airway against influenza infection.

#### 2.3.1. *Staphylococcus epidermidis*

*Staphylococcus epidermidis* is a dominant nasal commensal consistently associated with reduced influenza replication and improved mucosal antiviral defense. Its presence correlates with heightened epithelial antiviral readiness, preservation of barrier integrity, and reduced viral permissiveness at the site of entry. The molecular mechanisms underlying these effects are discussed in detail in section 3 [7].

#### 2.3.2. *Streptococcus oralis* and Commensal *viridans streptococci*

Recent work has shown that certain strains of *Streptococcus oralis* can enhance innate immune readiness and reduce viral burden in influenza challenge models [21]. These organisms may act as mucosal immune trainers, promoting balanced antiviral signaling without excessive inflammation. This effect contrasts with the pathogenic streptococci discussed below, highlighting the importance of strain-level characterization.

#### 2.3.3. *Dolosigranulum pigrum* and *Corynebacterium accolens*

These taxa co-occur frequently and are associated with stability of the upper airway microbiome and lower incidence of respiratory infection in infants and adults [16,19,22]. Mechanistically, *Corynebacterium* species may produce lipids that inhibit *S. pneumoniae* growth, while *Dolosigranulum* appears to contribute to epithelial barrier maintenance and homeostatic immune signaling [22].

### 2.4. Pathobionts and Microbe–Virus Synergy

Pathobionts are resident or transient microbes that do not cause disease under stable conditions but become pathogenic when the mucosal environment changes.

#### 2.4.1. *Streptococcus pneumoniae*

Influenza infection upregulates epithelial receptors and exposes basement membrane components, facilitating pneumococcal adherence and invasion. Pneumococcal neuraminidases remove sialic acids from airway mucus, altering viscosity and receptor accessibility, which can enhance influenza spread and bacterial colonization [12,23].

#### 2.4.2. *Staphylococcus aureus*

Certain *S. aureus* strains produce proteases and lipases that facilitate influenza hemagglutinin activation and promote viral replication, contributing to necrotizing or hemorrhagic pneumonia, particularly in otherwise healthy young individuals [13,14,24].

#### 2.4.3. Moraxella and Haemophilus

These genera expand during viral infection and are associated with inflammation, mucus thickening, and exacerbation of airway disease, especially in children and individuals with asthma or COPD [20,25].

### 2.5. Functional Interaction Pathways

Together, these interactions show that the airway microbiome is not neutral during influenza infection—it either raises resistance or increases vulnerability, depending on its structural and functional composition.



**Pathway**

**Microbial Influence**

**Effect on Influenza**
Interferon signalingCommensals prime IFN-λ responsesReduced viral replicationGlycan remodelingBacterial neuraminidases remove sialic acidsEnhanced bacterial adhesion; altered viral entryProtease landscapeProtease and antiprotease balance varies by speciesDetermines HA activation and viral infectivityBarrier and mucus functionCommensals stabilize junctions and cilia movementProtects against deep lung spread


## 3. Mechanistic Interactions Between the Airway Microbiome and Influenza Virus

Microbes in the airway influence the early stages of influenza infection, the intensity and character of the host immune response, and the risk of secondary bacterial disease. These effects arise from multiple interacting mechanisms, which can be broadly grouped into protective pathways, where commensals help the host resist viral replication and tissue injury, and exacerbating pathways, where pathobionts or dysbiosis facilitate viral spread and promote inflammatory damage.

### 3.1. Protective Mechanisms

Although *Staphylococcus epidermidis* has emerged as the most extensively characterized protective nasal commensal in influenza models, its antiviral effects are strain-dependent rather than species-universal. Only a subset of strains induces interferon-λ priming, antiprotease activity, or metabolic reprogramming, underscoring the importance of strain-level resolution when defining protective microbiome functions.

#### 3.1.1. Interferon-λ-Mediated Antiviral Priming

The first and most clearly defined protective mechanism is commensal-driven priming of interferon-λ (IFN-λ) signaling in the nasal epithelium. IFN-λ is the major antiviral cytokine at mucosal surfaces, where its receptor is primarily expressed on epithelial cells rather than immune cells. This allows strong antiviral responses without excessive inflammatory damage [6,7].

Nasal *Staphylococcus epidermidis* induces tonic IFN-λ expression*, leading to upregulation of interferon-stimulated genes (ISGs) such as OAS1, IFITM1/3, MX1, and ISG15, which block multiple steps of the influenza life cycle [7,8]. Importantly, this antiviral state is pre-emptive: it exists before viral exposure. Thus, the presence or absence of protective commensals can determine whether influenza replication is contained locally or allowed to spread into the lower respiratory tract [8].

Recent work demonstrated that depletion of these commensal-driven signals (for example, through antibiotics) significantly reduces early IFN-λ responses, resulting in higher viral titers and more severe lung pathology [5,26].

#### 3.1.2. Regulation of Protease Activity and Hemagglutinin Activation

Influenza virus infectivity requires that its hemagglutinin precursor (HA0) be cleaved into HA1 and HA2 by a host or microbial protease. The availability of these proteases determines whether influenza virions released from a cell are infectious and able to initiate further replication [13,27].

Commensals such as *S. epidermidis* increase expression of the antiprotease Serpine1, which inhibits serine proteases required for HA cleavage [9]. By reducing protease activity on the epithelial surface, commensals limit the activation of new virions, effectively reducing the local reproduction number of the virus at the entry point.

This mechanism is powerful because it does not require immune cell activation; it operates at the level of biochemical accessibility.

#### 3.1.3. Metabolic Reprogramming: Polyamine Depletion

Influenza replication is dependent on intracellular polyamines, which support viral RNA synthesis and ribonucleoprotein (RNP) complex assembly. A recent study demonstrated that *S. epidermidis* modifies host epithelial polyamine metabolism, reducing levels of spermine/spermidine and shifting the intracellular environment to a viral replication–limiting state [9]. This represents a metabolic defense strategy, conceptually similar to nutrient limitation in gut pathogen defense.

This discovery suggests that not only immune signaling but also cellular metabolism is shaped by airway commensals.

#### 3.1.4. Maintenance of Epithelial Barrier and Mucociliary Clearance

Commensals help maintain tight junction proteins (e.g., claudins, occludin, ZO-1) and support cilia beat coordination, both of which are necessary for clearing viral particles and inhaled bacteria [22,28]. When barrier integrity is preserved, influenza infection remains shallow and self-limited, rather than progressing into the lower respiratory tract where life-threatening pneumonia can develop.

#### 3.1.5. Protective Mechanisms Beyond *Staphylococcus epidermidis*

Protective effects against influenza are not restricted to *Staphylococcus epidermidis*. Certain viridans-group streptococci, including specific strains of *Streptococcus oralis*, have been shown to enhance epithelial antiviral readiness and reduce viral burden without inducing excessive inflammation. These organisms appear to promote balanced innate immune activation, potentially through tonic pattern-recognition signaling and modulation of epithelial gene expression.

Additional taxa associated with airway stability, such as *Dolosigranulum pigrum* and *Corynebacterium accolens*, are linked to reduced respiratory infection risk and may contribute indirectly by maintaining epithelial barrier integrity, producing antimicrobial lipids, and limiting expansion of pathobionts. While their influenza-specific mechanisms remain less well defined than those of *S. epidermidis*, their consistent association with microbial stability suggests an important supportive role in shaping a protective airway ecosystem [6,16,19,21,22].

### 3.2. Exacerbating Mechanisms

In contrast, dysbiosis and expansion of pathobionts during or before influenza infection increase viral infectivity and risk of secondary bacterial pneumonia.

#### 3.2.1. Bacterial Neuraminidases and Glycan Remodeling

Influenza virus binds to sialic acids on epithelial surfaces. Viral neuraminidase (NA) cleaves sialic acids to release new virions. However, pneumococcal neuraminidases (NanA, NanB, NanC) can also remove sialic acids, reshaping airway surfaces in ways that:

- Expose bacterial adhesion sites
- Reduce mucus viscosity
- Increase viral access to epithelial receptors

This creates a feedback loop: influenza promotes pneumococcal expansion, and pneumococcal activity enhances influenza spread [12,23,29].

A 2025 study further demonstrated that pneumococcal neuraminidase activity alters mucus viscoelastic structure, enabling denser and more persistent colonization, providing a strong mechanistic explanation for post-influenza bacterial superinfection [29].

#### 3.2.2. Bacterial Proteases That Activate Influenza Virus

While protective commensals reduce protease activity, certain pathobionts increase it. *Staphylococcus aureus* secretes V8 serine protease, staphopains, and Lipase 1, which enhance influenza viral entry and replication [13,14,24,30]. In severe co-infection, this leads to necrotizing or hemorrhagic pneumonia, particularly notable in cases of community-acquired MRSA in young adults without underlying disease [24,31].

This reveals an opportunistic cooperation between influenza and *S. aureus*, where each pathogen creates conditions that benefit the other.

#### 3.2.3. Influenza-Mediated Suppression of Phagocyte Function

Influenza virus transiently impairs the antibacterial functions of innate immune cells, particularly alveolar macrophages and neutrophils, creating a permissive window for secondary bacterial expansion. Although influenza infection induces robust recruitment of these phagocytes to the lung, viral infection paradoxically suppresses their antimicrobial capacity. Mechanistically, influenza reduces macrophage phagocytosis, reactive oxygen species production, and bacterial killing through interferon-dependent metabolic reprogramming and downregulation of scavenger and complement receptors [32].

Neutrophil dysfunction during influenza is characterized by impaired chemotaxis, reduced extracellular trap (NET) formation, and dysregulated degranulation, which together limit effective bacterial clearance while exacerbating tissue damage. Importantly, these defects persist beyond peak viral replication, overlapping temporally with the emergence of dysbiosis and expansion of pathobionts such as *Streptococcus pneumoniae*, *Haemophilus influenzae*, and *Moraxella* species. Thus, influenza-induced phagocyte paralysis represents a key immunological mechanism linking viral infection to microbiome destabilization and secondary bacterial pneumonia [20,25].

#### 3.2.4. Tissue Damage and Oxygenation Microenvironments

Influenza-induced epithelial injury and inflammation also reshape the physicochemical environment of the airway, particularly local oxygen availability, with important consequences for microbial ecology. Viral cytopathic effects, vascular leakage, and immune cell infiltration increase oxygen consumption and disrupt normal diffusion gradients, generating microaerophilic or hypoxic niches within inflamed airway tissue.

These altered oxygen microenvironments selectively favor facultative anaerobic bacteria, including streptococci and staphylococci, while disadvantaging obligate aerobes and protective commensals associated with microbial stability. In parallel, hypoxia-inducible factor (HIF) signaling in epithelial and immune cells modifies antimicrobial peptide expression, mucus composition, and iron availability, further influencing bacterial fitness. Together, influenza-driven changes in oxygen tension act as an ecological filter that promotes pathogen overgrowth and reinforces dysbiosis during and after viral infection [33]. This shift further enhances pathogen overgrowth and inflammation.

## 4. The Gut–Lung Axis and Systemic Microbiome Influence on Influenza

Although the airway microbiome directly shapes the local environment where influenza virus first establishes infection, the gut microbiome also influences disease outcomes through systemic immunometabolic signaling. This bidirectional communication is known as the gut–lung axis, in which metabolites, cytokines, and immune cells circulate between the intestinal and respiratory mucosae to coordinate host responses [34,35]. As a result, changes in gut microbial composition—due to diet, antibiotics, age, or illness—can modify pulmonary antiviral defenses, inflammatory responses, and tissue repair capacity during influenza infection.

### 4.1. Short-Chain Fatty Acids as Key Immune Mediators

Short-chain fatty acids (SCFAs), particularly acetate, propionate, and butyrate, are produced by microbial fermentation of dietary fiber in the colon. These metabolites reach the lungs through the circulation, where they influence epithelial and immune cell behavior [36].

Recent mechanistic studies have demonstrated that acetate plays a particularly important protective role in viral respiratory infections. In mouse models of influenza, acetate:

- Strengthens tight junctions by enhancing expression of claudins and occludin
- Reduces epithelial permeability and limits viral spread to deeper lung tissue
- Modulates inflammatory responses to prevent excessive tissue damage
- Improves clinical outcomes and survival following viral challenge [15]

These effects occur without suppressing antiviral immunity. Instead, acetate supports a controlled immune response, one that clears the virus efficiently without triggering the damaging hyperinflammation that contributes to severe influenza pneumonia.

Dietary fiber intake directly influences acetate availability, suggesting a link between nutritional patterns and respiratory disease resilience [37].

### 4.2. Effects on Innate and Adaptive Immune Function

SCFAs also influence the function of multiple immune cell populations relevant to influenza defense: **Immune Cell Type****Influence of SCFAs****Functional Effect During Influenza**Alveolar macrophagesEnhanced metabolic fitness and phagocytic efficiencyFaster clearance of viral particles and apoptotic cellsNeutrophilsPromotion of balanced antimicrobial responsesReduced tissue-destructive inflammationRegulatory T cells (Tregs)Increased differentiation and activationPrevention of overwhelming inflammationDendritic cellsModulation of antigen presentation capacityCan influence influenza vaccine responsiveness

These effects shape the quality rather than the magnitude of the immune response. A well-regulated response clears the virus efficiently, while minimizing lung injury and secondary bacterial susceptibility.

### 4.3. Influence of Antibiotics and Intestinal Dysbiosis

Evidence linking antibiotic-associated gut dysbiosis to worsened influenza outcomes derives largely from models using broad-spectrum antibiotic regimens, which induce profound and sustained depletion of commensal bacteria and their metabolites. In murine studies, combinations targeting both Gram-positive and Gram-negative organisms markedly reduce short-chain fatty acid availability, blunt type I and type III interferon responses, and delay viral clearance, leading to increased lung injury and mortality [5,26,38]. In contrast, narrower-spectrum or short-duration antibiotic exposure appears to cause more transient microbiome disruption, with less pronounced impairment of antiviral immunity, although this distinction has not been systematically evaluated in humans.

Importantly, the timing and duration of antibiotic exposure are critical: antibiotic treatment preceding or overlapping early influenza infection has the strongest negative impact on antiviral defense, whereas exposure after viral clearance may primarily affect recovery and susceptibility to secondary bacterial colonization. Clinically, these findings suggest that unnecessary broad-spectrum antibiotic use during influenza season—particularly in viral syndromes without evidence of bacterial infection—may inadvertently increase disease severity by disrupting gut–lung immune crosstalk.

### 4.4. Tryptophan Metabolism and Epithelial Repair

In addition to SCFAs, the gut microbiome regulates metabolism of the amino acid tryptophan into:

- Indoles (microbial pathway) → enhance epithelial repair and reduce inflammation
- Kynurenine (host pathway) → suppresses adaptive immunity under inflammatory stress

A balanced microbiome promotes indole production. In contrast, inflammatory dysbiosis increases kynurenine, which has been linked to:

- Impaired antiviral T cell responses
- Delayed mucosal recovery after infection
- Prolonged airway vulnerability to bacterial colonization [39]

This is particularly relevant in older adults and individuals with metabolic syndrome, who often show both reduced indole-producing gut microbes and poorer influenza outcomes.

### 4.5. Integrating the Airway and Gut Microbiome in Influenza Pathogenesis

The airway and gut microbiomes do not act independently. Instead:

- The gut microbiome sets systemic immune tone, influencing the readiness and resilience of lung tissue.
- The airway microbiome determines local antiviral conditions at the precise site of exposure.

Together, they form a two-layered defense system:

1. Systemic preparedness (immune conditioning via gut microbial metabolites)
2. Local antiviral resistance (commensal-driven IFN-λ priming and barrier protection in the nasal mucosa)

When both layers are intact, influenza is often mild and self-limiting.

When either layer is disrupted, viral replication is more robust and lung pathology more severe.

When both layers are disrupted (e.g., antibiotics + airway dysbiosis), the risk of secondary bacterial pneumonia is highest.

## 5. Translational and Therapeutic Perspectives

Most microbiome-targeted strategies discussed below are currently supported by preclinical evidence from animal models, in vitro airway systems, or early-phase translational studies, with limited direct clinical data in humans. Where available, we distinguish between preclinical proof-of-concept and interventions that have entered early clinical evaluation.

Growing mechanistic insight into how the airway microbiome modulates influenza infection has led to the development of microbiome-informed therapeutic strategies. These approaches are not intended to replace vaccines or antiviral drugs, but to reinforce mucosal defenses, reduce viral replication at the initial site of infection, and decrease the likelihood of secondary bacterial complications. Three main translational avenues are currently under investigation: (1) intranasal live biotherapeutic products (LBPs), (2) metabolite-based or diet-driven interventions, and (3) therapies targeting microbial and host enzymatic interactions that influenza exploits.

### 5.1. Intranasal Live Biotherapeutic Products (LBPs)

To date, human data for airway-targeted LBPs remain limited to early safety and feasibility considerations, and robust efficacy data from controlled clinical trials are not yet available.

LBPs are intentionally selected beneficial bacteria delivered directly into the nasal cavity, where influenza infection typically begins. Unlike oral probiotics, which must survive gastrointestinal barriers and systemic distribution, intranasal LBPs act locally, at the epithelial surface where commensal–virus interactions occur. Although intranasal live biotherapeutic products are designed to reinforce mucosal defenses rather than establish invasive infection, their safety profile requires careful consideration, particularly in immunocompromised individuals and patients with chronic lung or systemic disease. To date, most preclinical and early translational studies of airway-targeted LBPs have been conducted in immunocompetent hosts, and long-term safety data in high-risk populations remain limited.

Potential concerns include unintended persistence or overgrowth of administered strains, disruption of existing microbial equilibrium, and rare risk of opportunistic infection in individuals with impaired mucosal barriers or immune surveillance. For these reasons, current LBP development emphasizes strict strain selection, exclusion of virulence and antibiotic resistance determinants, and strategies favoring transient colonization rather than permanent microbiome alteration. Until more clinical data are available, the use of LBPs in severely immunocompromised patients or those with advanced chronic respiratory disease should be approached cautiously and evaluated within controlled clinical trial settings.

#### 5.1.1. *Staphylococcus epidermidis*–Based LBPs

As described in earlier sections, specific strains of *Staphylococcus epidermidis* promote interferon-λ-mediated antiviral priming, enhance Serpine1-dependent protease inhibition, and induce polyamine depletion in nasal epithelial cells, collectively restricting influenza virus replication [7,8,9].

These properties make *S. epidermidis* an appealing first-generation LBP candidate.

However, *S. epidermidis* is genetically diverse, and strain-level selection is essential. Some strains carry genes involved in biofilm formation or immune evasion that would not be appropriate for therapeutic deployment [40]. Current development pipelines therefore include:

- Whole-genome screening to eliminate strains harboring toxin or superantigen genes
- Testing of epithelial compatibility in organoid and air–liquid interface (ALI) culture models
- Transient colonization strategies, where bacteria act locally without permanent microbiome alteration

This aligns with the regulatory framework for LBPs defined by the U.S. FDA and European Medicines Agency, where stability, genetic traceability, and absence of virulence factors are required for approval [41].

#### 5.1.2. Commensal Streptococci LBPs, Including *Streptococcus oralis*

Certain strains of *Streptococcus oralis* have been shown to enhance mucosal antiviral phasing and inhibit influenza replication in vivo [21]. These strains appear to promote a balanced immune activation, distinct from the strong IFN-λ priming attributed to *S. epidermidis*, suggesting that combination LBP formats could target multiple antiviral pathways at once.

A key consideration is that commensal streptococci exist along a continuum with pathogenic streptococci, including *Streptococcus pneumoniae*.

Therefore, capsule gene absence, pneumolysin negativity, and non-invasive phenotypes must be confirmed before translational advancement [42].

#### 5.1.3. Spore-Based LBPs (e.g., *Bacillus* spp.)

Spore-forming bacteria such as *Bacillus subtilis* and *Bacillus coagulans* are being tested intranasally and have shown potential to:

- Reduce symptom severity
- Accelerate viral clearance
- Limit bacterial superinfection risk in acute viral respiratory infections [43]

Spores are stable, inexpensive, and robust, making them suitable for population-wide, seasonal preventive use. Their antiviral mechanisms may include trained innate immunity, in which epithelial and myeloid cells exhibit enhanced baseline readiness following microbial exposure [44].

However, unlike *S. epidermidis*, the precise mechanistic pathways of spore-based LBPs against influenza require additional clarification.

### 5.2. Microbial Metabolite and Dietary Interventions

#### 5.2.1. Acetate-Based Therapies

Protective effects of acetate during influenza infection have been demonstrated predominantly in mouse models and epithelial systems, where acetate preserves barrier integrity and limits lung injury. While these findings support translational interest, clinical data directly linking acetate supplementation to improved influenza outcomes in humans are currently lacking.

Acetate protects the lung during influenza infection by maintaining tight junction integrity and reducing inflammatory epithelial damage, without impairing viral clearance [15]. Consequently, two therapeutic formats are being explored:

1. Systemic acetate supplementation (dietary fiber fermentation support, SCFA derivatives)
2. Topical/intranasal acetate delivery, which may provide direct barrier protection in the upper airway

Given its safety and metabolic familiarity to the host, acetate is a promising adjunctive therapy, particularly for older adults and individuals with metabolic disease, who are more prone to exaggerated inflammatory lung injury.

#### 5.2.2. Tryptophan–Indole Enhancement

Gut microbiota that convert tryptophan into indole derivatives support mucosal repair and maintain controlled inflammation [39]. Microbial or small-molecule enhancers of the indole–aryl hydrocarbon receptor (AhR) pathway are being evaluated as recovery-phase therapies to reduce post-influenza lung damage and susceptibility to bacterial colonization.

### 5.3. Targeting Host–Microbe–Virus Enzymatic Interactions

#### 5.3.1. Neuraminidase-Targeted Approaches

Influenza infection depends on sialic acid recognition. One therapeutic strategy uses recombinant sialidases (e.g., DAS181) that remove viral binding sites on epithelial surfaces, reducing influenza replication and viral shedding [45]. DAS181 has shown efficacy against multiple influenza subtypes in clinical testing, including cases resistant to oseltamivir.

However, removal of sialic acids may also affect bacterial colonization patterns, necessitating controlled dosing and short-duration application.

#### 5.3.2. Modulating the Protease Environment

Because membrane proteases such as TMPRSS2 activate influenza HA, protease inhibition can reduce infectivity.

The serine protease inhibitor camostat mesylate has demonstrated reductions in influenza entry and replication in airway epithelial models [46].

At the same time:

- Commensals increase antiprotease tone (e.g., Serpine1)
- Pathobionts increase protease secretion (e.g., *S. aureus* V8 protease, staphopains [13,30])

Thus, protease environment modulation represents a shared mechanistic interface where microbial therapy and pharmacologic therapy can operate synergistically.

### 5.4. Integrative Therapeutic Framework

Rather than functioning independently, conventional influenza interventions and microbiome-targeted strategies are complementary: **Approach****Primary Effect****Best Use Window**VaccinesReduce susceptibility and disease severityPre-season and high-risk groupsAntiviralsLimit viral replication after infectionWithin first 48 hLBPsStrengthen mucosal antiviral preparednessPre-exposure or very early post-exposureSCFA/acetate therapyPreserve epithelial barrier and reduce tissue injuryEarly infection and high-risk patientsProtease/neuraminidase modulationRestrict viral entry and activationEarly infection and co-infection risk cases

The most realistic clinical future involves layered protection, in which vaccination is supplemented with microbiome stabilization strategies during influenza season.

Collectively, these approaches illustrate a translational pipeline in which mechanistic insights from preclinical models are beginning to inform early human investigation, but widespread clinical application will require carefully designed trials to establish safety, efficacy, and microbiome stability.

## 6. Challenges, Knowledge Gaps, and Future Research Directions

While the role of the airway microbiome in modulating influenza outcomes is increasingly well-supported, several practical barriers currently limit translation into clinical practice. Addressing these gaps will be essential for advancing microbiome-based prevention and treatment strategies.

### 6.1. Strain-Level Specificity and Reproducibility

Microbiome effects on influenza are not uniform at the species level. For example, only certain strains of *Staphylococcus epidermidis* induce IFN-λ antiviral priming, and only a subset of *Streptococcus oralis* strains exhibit mucosal immune-supporting properties [7,8,9,21]. Meanwhile, strains of *S. epidermidis* associated with prosthetic infections or skin dysbiosis are not suitable for intranasal delivery [40].

Because of this, selecting microbes for therapeutic development requires:

- Whole-genome sequencing to screen out virulence genes
- Confirmation of non-biofilm-forming, non-invasive phenotypes
- Functional verification in airway epithelial and mucosal immune models

This represents a shift away from taxonomy-based selection toward function-first strain characterization—a standard now recognized in LBP regulatory guidance [41].

### 6.2. Sampling and Methodological Variability

The airway is a low-biomass microbial environment, making it susceptible to contamination during collection and sequencing. Differences in:

- Sampling site (anterior nares vs. nasopharynx),
- DNA extraction methods,
- Sequencing depth,
- Bioinformatics pipelines

can produce conflicting conclusions between studies [20,34,47].

Improved standardization of airway sampling and analytic procedures will be necessary to compare results across research centers and to conduct reproducible clinical trials.

### 6.3. Bridging Mechanistic Evidence to Human Clinical Validation

Most mechanistic understanding derives from murine models, in vitro airway culture, or ex vivo mucosal explants, which cannot fully replicate human mucosal complexity, immune memory, viral exposure history, or environmental variation [35,48].

Controlled human colonization studies—where defined commensal strains are delivered intranasally under close monitoring—represent the next major step. Ethical precedents already exist, including controlled pneumococcal colonization models used safely in adult volunteers [49], which can inform clinical testing of commensal LBPs.

### 6.4. Timing and Dosing Challenges

Microbiome-mediated antiviral protection works best before or very early in influenza infection. This raises practical questions:

- Should LBPs be administered only in high-risk groups, or seasonally to the general population?
- Is stable colonization desirable, or is transient mucosal conditioning safer and sufficient?
- Should metabolic adjuncts (e.g., acetate) be taken continuously or at first symptom?
- These timing variables remain largely untested in humans.

### 6.5. Interactions with Vaccines and Antivirals

Because both vaccines and LBPs act on mucosal immune pathways, their interaction must be carefully evaluated. For example:

- IFN-λ priming could theoretically enhance vaccine-induced responses, by improving antigen presentation and T cell recruitment.
- However, excessive epithelial interferon signaling could transiently reduce vaccine antigen uptake.

Similarly, neuraminidase-targeting interventions (e.g., DAS181) may alter bacterial attachment dynamics, requiring appropriate dosing windows to avoid unintended dysbiosis [45].

## 7. Conclusions

The airway microbiome is now recognized as a critical determinant of influenza infection outcomes, influencing viral replication at the point of entry, shaping mucosal immune activation, and determining the likelihood of secondary bacterial pneumonia. Protective commensals, including *Staphylococcus epidermidis*, *Streptococcus oralis*, *Dolosigranulum pigrum*, and *Corynebacterium accolens*, promote interferon-λ signaling, stabilize the epithelial barrier, and modify metabolic and protease environments to make the nasal mucosa less permissive to viral infection. In contrast, expansion of pathobionts such as *Staphylococcus aureus*, *Streptococcus pneumoniae*, *Haemophilus influenzae*, and *Moraxella catarrhalis* during influenza contributes to severe disease, enhanced viral infectivity, and bacterial superinfection.

Mechanistic clarity has now translated into therapeutic opportunity. Intranasal live biotherapeutics, acetate-mediated barrier support, and host–microbe enzymatic modulation represent promising strategies to complement vaccines and antiviral drugs. These approaches aim not to eliminate influenza virus directly, but to reinforce the mucosal environment, improving disease resilience and reducing the likelihood of complications.

Looking forward, progress will depend on strain-level microbial characterization, standardized airway microbiome profiling, and well-designed human trials evaluating preventive and early-intervention strategies. With these advances, microbiome-guided therapy may become a core component of influenza preparedness and seasonal respiratory disease management.

## Data Availability

No new data were created or analyzed in this study. Data sharing is not applicable to this article.
